# PD-1 Inhibitor Maintenance Therapy Combined Iodine-125 Seed Implantation Successfully Salvage Recurrent Cervical Cancer after CCRT: A Case Report

**DOI:** 10.3390/curroncol28060387

**Published:** 2021-11-09

**Authors:** Guangchao Wei, Fuxin Guo, Ang Qu, Weijuan Jiang, Yuliang Jiang, Junjie Wang, Ping Jiang

**Affiliations:** 1 Institute of Medical Technology, Peking University Health Science Center, Beijing 100000, China; 1911210524@pku.edu.cn; 2 Department of Radiation Oncology, Peking University 3rd Hospital, Beijing 100191, China; guofuxinpku@bjmu.edu.cn (F.G.); 0463180457@bjmu.edu.cn (A.Q.); 0263177456@bjmu.edu.cn (W.J.); yuliangjiang@bjmu.edu.cn (Y.J.); junjiewang@pku.edu.cn (J.W.)

**Keywords:** recurrent cervical cancer, iodine-125 seed implantation, PD-1, immune checkpoint inhibitors, low-dose-rate

## Abstract

Cervical cancer is the fourth most common cancer in females worldwide. Patients with stage III and IV cervical cancer based on the Federation of Gynecology and Obstetrics (FIGO) classification have higher recurrence rates. Because of organs at risk (OAR) protection and the low indication rate of salvage surgery, the choice of treatment is always challenging. Systemic chemotherapy is palliative and can be performed in conjunction with surgery or radiotherapy; however, it has no significant benefit to survival. Brachytherapy and stereotactic body radiotherapy (SBRT) are characterized by extremely high radiation doses applied to tumor cells while sparing the normal tissues. Several studies have investigated the efficacy of these technologies in recurrent cervical cancer and showed promising results. The immune checkpoint inhibitors approach was also investigated and showed promising results too. Herein, we report a case of a patient with cervical cancer that recurred five months after adjuvant chemotherapy and concurrent chemoradiotherapy. The disease prognosis after interstitial implantation brachytherapy (IIB) was determined. Then, the patient underwent radioactive 125I-seed implantation combined with PD-1 inhibitor treatment. The patient exhibited a partial response after seed implantation, and up to now, the duration of this partial response was 24 months.

## 1. Introduction

Cervical cancer is the fourth most common cancer in females worldwide [1]. According to the International Agency for Research on Cancer projections, 528,000 new cases occurred in 2012, and in the same year, cervical cancer was responsible for 266,000 deaths, accounting for 7.5% of all female cancer deaths. Its recurrence rates in patients with International Federation of Gynecology and Obstetrics (FIGO) stages Ib, IIa, IIb, III, and IVa are 10%, 17%, 23%, 42%, and 74%, respectively, and 80% of cervical cancer recurrences occur within two years after the initial treatment [2,3]. The organs at risk (OARs) are proximate to the tumor site, and re-irradiation will increase the risk of damage to normal tissues [4]. Since about 70% of patients with recurrent cervical cancer have undergone radiotherapy, the treatment option is always challenging.

Selective anti-angiogenic therapy by tyrosine kinase inhibitors may represent a novel therapeutic tool, which was used alone or in combination with chemotherapy [5]. The vascular endothelial growth factor (VEGF) and the epidermal growth factor receptor (EGFR) is the most commonly selected proangiogenic factors for anti-angiogenic therapy, and the US Food and Drug Administration (FDA) has approved an anti-VEGF targeted therapy in combination with chemotherapy for patients with metastatic, persistent, or recurrent cervical cancer, and several targeted therapies are still investigated [5,6].

Poly ADP-ribose polymerase (PARP) is an intracellular protein involved in the repair of single-stranded DNA breaks, which was used by tumor cells to repair errors in DNA replication in the setting of high mutational burden that is a hallmark of malignancy [7,8,9]. A phase I trial indicated that PARP inhibitor was safe and feasible [10].

Early reports have implicated PI3KCA somatic mutations suggesting that mTOR-targeted agents should be explored in cervical cancer [11]. Programmed death-ligand 1 (PD-L1) inhibitor is an immune checkpoint inhibitor with high specificity for binding to a programmed cell death 1 (PD-1) receptor. It was approved by the FDA for patients with recurrent or metastatic cervical cancers [12]. Anti-cytotoxic T lymphocyte-associated antigen 4 (CTLA-4) is another target for therapy being studied in a variety of malignancies, including cervical cancer [13,14]. Several phase I or II trials are now evaluating its safety and efficacy in the treatment of advanced cervical cancer [5].

Radioactive 125I seed implantation (RISI) is a kind of low-dose-rate (LDR) brachytherapy, which was characterized by extremely high steep dose-fall off and was initially a standard treatment option for men with localized prostate adenocarcinoma. A low-dose-rate (LDR) with the computer tomography (CT) guidance and 3-dimensional (3D)-printing non-coplanar template (PNCT) assistance, RISI can achieve an accurate dose delivery and spare the normal tissues. Up to now, 3D PNCT-assisted RISI has presented promising results in the salvage treatment of several kinds of recurrent tumors [15,16,17,18] and has been referenced in the National Comprehensive Cancer Network guidelines for the management of a locally recurrent rectal cancer [19]. Meanwhile, existing studies have preliminarily confirmed the efficacy and safety of RISI in the treatment of cervical cancer [16,20,21]. Meanwhile, recent studies indicated that radiotherapy induces immunomodulatory effects [22,23,24,25]; therefore, we expect the combination of PD-1 immune checkpoint inhibitor and RISI radiotherapy would improve prognosis compared with monotherapy and presented this case.

## 2. Materials and Methods

A 46-year-old woman with recurrent cervical cancer was referred to our hospital. The patient was diagnosed with cervical cancer on 10 March 2018. Based on imaging and pathologic examination, the patient was diagnosed with FIGO stage IIIB (T4N1M0) cervical cancer. The tumor invaded the left urethra, and the patient had left kidney failure with hydronephrosis. Initially, the patient received concurrent chemoradiotherapy (CCRT). Before the treatment, the squamous cell carcinoma antigen (SCC-Ag) was 5.3 μg/L (normal value: ≤1.5 μg/L). External beam radiotherapy (EBRT) and planning target volume (PTV) included the primary tumor, cervix, uterine, upper vagina, and pelvic lymphatic drainage area. The pelvic lymph node’s gross target volume (PGTVnd) includes the metastatic pelvic lymph nodes, while the planning target volume-1 (PTV1) includes the parametrium lymph nodes. The prescription dose of PGTVnd was 60 Gy, 2.14 Gy/28 f, and that of PTV1 was 60 Gy, 2.14 Gy/28 f. High-dose-rate (HDR) intracavitary brachytherapy was commenced when EBRT was delivered. A prescription dose at point A involved 30 Gy/5 f, 2 f/w. About 30 mg of cisplatin was infused weekly during radiotherapy.

**Figure 1:**
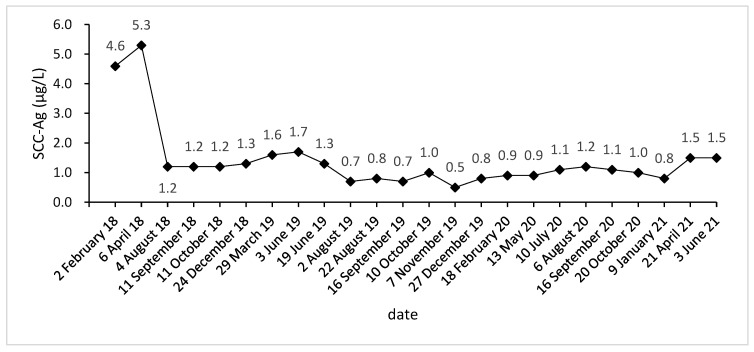
SCC-Ag level acquired before, during, and after treatment.

**Figure 2:**
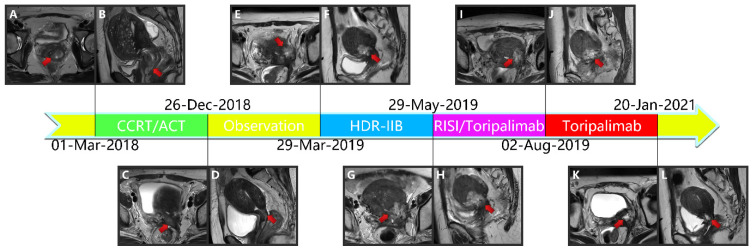

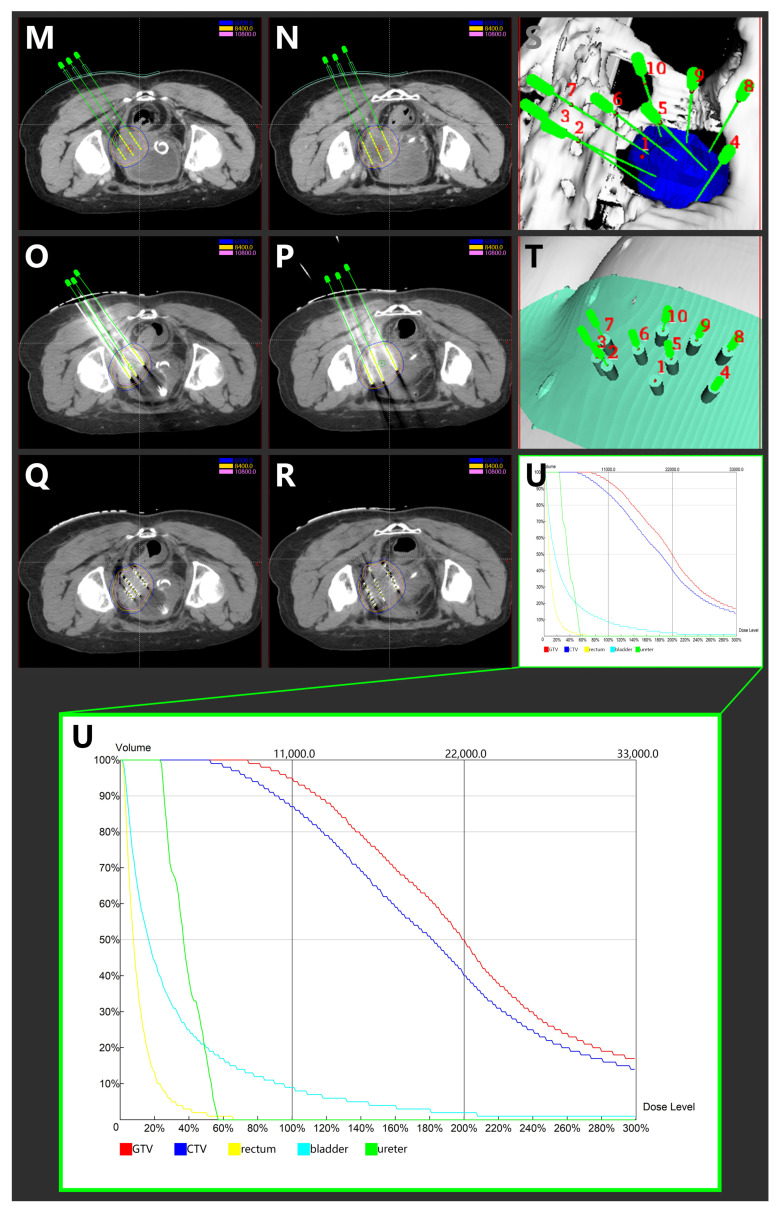
(**A**,**B**) Magnetic resonance imaging taken before CCRT in T2-weighted sequence. (**C**,**D**) Magnetic resonance imaging taken after CCRT and ACT in T2-weighted sequence. (**E**,**F**) Magnetic resonance imaging depicting the cervical cancer recurrence after CCRT and ACT in T2-weighted sequence. (**G**,**H**) Magnetic resonance imaging depicting the cervical cancer recurrence after CCRT, ACT, and HDR-IIB in T2-weighted sequence. (**I**,**J**): Magnetic resonance imaging taken after 3D-PNCT assisted RISI in T2-weighted sequence. (**K**,**L**) Magnetic resonance imaging taken after 3D-PNCT assisted RISI in T2-weighted sequence. (CCRT, concurrent chemoradiotherapy; ACT, adjuvant chemotherapy; HDR, High-dose-rate; IIB, interstitial implantation brachytherapy; 3D, 3-dimensional; PNCT, printing non-coplanar templates; RISI, radioactive 125I seed implantation). (**M**,**N**) Portion of pre-plan images. (**O**,**P**) Portion of real-time dose optimization images. (**Q**,**R**) Portion of post-plan images. (**S**,**T**) 3D-PNCT assisted RISI reconstruction visualization for recurrent cervical cancer. (**U**) Dose-volume histogram of 125I seeds. (3D, 3-dimensional; PNCT, printing non-coplanar templates; RISI, radioactive 125I seed implantation).

Due to vaginal bleeding, anemia, and hypothrombocytopenia, CCRT was terminated within 84 days. After CCRT, the patient received adjuvant chemotherapy (ACT) for three cycles (Docetaxel + Carboplatin) and presented grade 3 leukocyte reduction and grade 2 anemia. The SCC-Ag during ACT was 1.2, 1.2, and 1.3 μg/L (Figure 1). After the three cycles, the tumor response was assessed as a partial response (PR) (Figure 2A–L).

Approximately five months after the ACT, SCC-Ag increased to 1.6 μg/L (29 March 2019), and the MRI also hints at an increased focus and pelvic wall invasion, suggesting tumor recurrence. Then, the patient underwent a template-assisted CT-guided biopsy that diagnosed the recurrence of cervical cancer. Immunohistochemistry: Ki-67 (50%+), CK (+), P40 (+), CD34 (−), and CD31 (−).

Upon the diagnosis of recurrent cervical cancer, the patient underwent HDR interstitial implantation brachytherapy (IIB). The prescription dose was 36 Gy/6 Gy/6 f 2 f/w. The actual completion of HDR-IIB was four fractions due to poor response. The MRI (29 May 2019) scan after four times HDR-IIB hints at progressive disease (PD). The volume of the tumor increased from 2.9 × 2.4 × 1.5 cm to 3.6 × 3.5 × 2.6 cm, and DWI-MRI exhibited an irregular hyperintense signal. The SCC-Ag was 1.7 μg/L on 3 June 2019 and 1.3 μg/L on 19 June 2019. Based on the MRI and SCC-Ag results, IIB did not achieve a curative effect as we expected.

Subsequently, the patient underwent ureteral stent placement on 13 June 2019 and received CT-guided and 3-dimensional (3D)-PNCT-assisted radioactive 125I seed implantation (RISI) (20 June 2019), combined with PD-1 inhibitor treatment (240 mg toripalimab q21d).

The 3D-PNCT-assisted RISI was carried out following the process mentioned subsequently. The patient received a CT simulation with contrast and 5-mm slice thickness before RISI. Then, the CT simulation images were uploaded to the brachytherapy treatment planning system (BT-TPS, KLSIRPS-3D; Beijing Tianhang Kelin Technology Development Inc., Beijing, China) for pre-plan, target volume, and OAR delineation (Figure 2M–U). The prescription dose was 110 Gy, and the pre-plan evaluation was GTV D90 = 144.5 Gy, GTV V100 = 97.6%, and CTV D90 = 109.4, CTV V100 = 89.0%. The pre-plan dataset was used for digital modeling and the printing of individualized 3D-PNCTs, which included the biological surface characteristics of the seed implantation area, the *X*-axis and *Y*-axis laser lines, a registration mark, and the information about the simulated needle path. RISI was carried out under spinal anesthesia. When the patient and 3D-PNCT were re-set up, single-use needles were inserted into the target lesion under CT guidance. A Mick applicator was used to implant the seeds. After seed implantation, a CT scan was performed again to check the distribution of actual 125I seeds in the targets, and additional seeds would be implanted if the distribution of 125I seeds was not satisfactory. The CT image dataset was transferred to the BT-TPS for post-planning dose evaluation [16,20]. The evaluated doses were GTV D90 = 126.9 Gy, GTV V100 = 94.7%, and CTV D90 = 100.2 Gy, CTV V100 = 87.0%.

The injection of 240 mg toripalimab was performed for the first time one day after seed implantation. After two cycles of PD-1 inhibitor treatment, the SCC-Ag was 0.7 μg/L on 2 August 2019. Meanwhile, the MRI acquired on 2 August 2019 reported that the tumor bed volume shrank from 3.6 × 3.5 × 2.6 cm to 3.1 × 3.3 × 2.6 cm, and the T2-weighted image signal intensity decreased significantly, which was considered as a PR. After that, the patient accepted another 15 cycles of PD-1 inhibitor treatment during the next 15 months. At this stage, the highest level of SCC-Ag was 1.2 μg/L. Additionally, the patient underwent six MRI scans and these MRIs hint at PR.

## 3. Results

After seed implantation and PD-1 inhibitor treatment, no evidence hints towards tumor recurrence or metastasis up to now. The patient exhibited a complete response (CR) after seed implantation. The duration of this CR was at least 24 months, meanwhile no grade 3 or 4 serious adverse events occurred after RISI.

## 4. Discussion

The recurrence rates of cervical cancer are 10%, 17%, 23%, 42%, and 74% for FIGO stages Ib, IIa, IIb, III, and IVa, respectively [2,3]. The main methods to treat recurrent cervical cancer are surgery, systemic chemotherapy, SBRT, HDR brachytherapy, EBRT (IMRT/VMRT), and chemotherapy; however, due to their dose limitation for OARs and other reasons, EBRT and surgery are often not selected [26]. The indication of salvage surgery is limited to only 1.5–7% of patients with recurrent disease [27]. Systemic chemotherapy is basically palliative and can be performed in conjunction with surgery or radiotherapy. However, according to the result of the Royal Marsden hospital, second-line systemic therapy, including targeted agent monotherapy and chemotherapy, did not show significant benefits for survival, the progression-free survival (PFS) for second-line therapy was only 3.2 months, and the median overall survival (OS) was 9.3 months, meanwhile, the objective response rate to second-line therapy was 13.2% [28]. Other studies also did not show significant benefits for survival, which is still less than 12 months for most patients [3,29]. RISI is characterized by extremely high radiation dose applied to tumor cells while sparing the normal tissues, so it might be a good selection. Initially, RISI is used to treat primary prostate cancer [30,31,32,33]. Accumulating evidence showed its good efficacy in various recurrent cancers, including rectal cancer, head and neck cancer, and pancreatic cancer [17,34,35,36,37,38]. Moreover, our previous studies demonstrated that 3D-PNCT-assisted CT-guided RISI is a safe, effective, and minimally invasive modality to treat recurrent cervical cancer after radiotherapy [16,20]. Other centers showed similar results [21].

According to our previous study [16], which included 111 lesions from 103 patients, the response (complete response, CR/PR/stable disease, SD) of tumors after RISI was 99.1%. The local control (LC) for one year and three years was 87.4% and 75.1%, respectively. For patients with D90 ≥ 130 Gy for one year and three years, the LC was 98.6% and 95.8%, respectively. The PFS for one year and three years was 56.1% and 17.2%, respectively. Additionally, the overall survival for one year and three years was 68.1% and 20.8%, respectively; the median of the overall survival was 17 months. Radiation-related grade 2 early adverse events include nausea (1.0%), diarrhea (1.9%), and pollakiuria (1.0%). Grade 3 early adverse events included diarrhea (1.0%), and there were no grade 4/5 early nor 2/3/4/5 later adverse events reported, except in two patients with rectovaginal fistula (1.9%).

SBRT is a novel technology with dose characteristics similar to RISI. SBRT, with or without chemotherapy, was reported to treat recurrent or metastatic cervical cancers with a 2-year LC rate of 43–82.5% [39,40] and a 5-year LC rate of 78.8% [40]. Meanwhile, the 2-year and 5-year OS was 43–57.5% [39,40] and 32.9% [40], respectively. About 35% of patients experienced mild acute toxicity rate during and three months after SBRT [39], and 2–13% of patients had a fistula six months after SBRT (grade 4) [39,40]. All in all, the sample size of SBRT for recurrent cervical cancers is small or assorted.

As an alternative, some researchers investigated the effect and toxicity of the HDR-IIB technique. The objective response rate (ORR) was 100% to 76.9% [41,42,43,44], while the CR rate was 59.6% to 100% [41,42,43,44]. According to various studies, the 1-year OS was 71% [41], the 2-year LC/PFS/OS was 51.3%/20%/60.8% [42], the 3-year LC/PFS/OS was 45%/42%/68% [43], and the 5-year LC/PFS/OS was 51.3%/20.0%/30–52% [41,42]. The median OS after recurrence was 9.2 months to >60 months [41,42,43,44,45]. Recent studies also showed that patients had longer OS. The frequency of grade 3/4 toxicities was 25% to 33% [41,42,44]. About 17.3% to 31.1% of patients exhibited any kind of fistula [41,42,44]. In general, the sample size was also small and included different primary treatments.

From the Cancer Genome Atlas Network, detailed genomic analyses revealed the amplification of programmed death-ligand 1/2 (PD-L1/2) in cervical cancer tissues, supporting the immune checkpoint inhibitors approach [46], and it is related to poor clinical prognosis [47,48,49,50,51]. The KEYNOTE-158 study is a multicohort, single-arm, open-label, phase 2 study assessing the pembrolizumab monotherapy in patients with a solid tumor, which included 98 cervical cancer patients who progressed during the therapy or were intolerant to one or more lines of standard therapy. The ORR was 12.2%, with three CR and nine PR. All 12 responses were in 82 patients with PD-L1-positive tumors, with an ORR of 14.6%. About 11 of 77 patients previously received one or more lines of chemotherapy for recurrent or metastatic diseases. In addition, 18 out of 98 patients, 15 out of 82 PD-L1-positive patients, 13 of previously treated patients, and 3 of 15 PD-L1-negative patients had SD, leading to a disease control rate of 30.6% in the total population and 32.9% in the PD-L1-positive tumor population. The median OS and 1-year OS rates were 9.4 months/41.4% and 11 months/47.3% for the total and PD-L1-positive tumor population. Twelve (12.2%) patients experienced one or more grade 3 or 4 events [12]. When the patients were stratified according to tumor mutational burden (TMB), the median OS was 16.7 months for patients with high TMB and 9.4 months for patients without high TMB in the efficacy population group [52]. In another phase I/II clinical trials of nivolumab monotherapy on 19 recurrent or metastatic cervical cancer patients (NCT02488759), the ORR was 26.3% (ORR = 5, CR = 3, PR = 2, SD = 8, PD = 6), the median OS was 21.9 months, the 1-year OS and 2-year OS were 77.5% and 49.8%, respectively [53]. Toripalimab is a humanized IgG4K monoclonal antibody specific for human PD-1; the international, double-blind, phase 3 trial (NCT03581786) indicated that the addition of toripalimab to Gemcitabine-cisplatin (GP) chemotherapy as a first-line treatment for patients with recurrent or metastatic nasopharyngeal carcinoma provided superior prognosis compared to GP alone and with a manageable safety profile. Median PFS for Toripalimab arm and placebo arm was 11.7 months versus 8.0 months, and the hazard ratio (HR) = 0.52. Up to 18 February 2021, a 40% reduction in risk of death was observed in the toripalimab arm (HR = 0.603) [54].

It has been demonstrated that radiotherapy would influence the immune microenvironment and immune system [22,55,56,57]. Radiotherapy exerts its cytotoxic mitotic effects on tumor cells through DNA damage. In the past, it was viewed as an immunosuppressant [58,59]. Recent studies have demonstrated that radiotherapy will induce immunomodulatory effects through the tumor microenvironment and upregulation of the inflammatory cascade [22,23,24,25]. The ideal combination of immunotherapy and radiotherapy should overcome the resistance mechanisms. Chemoradiation increases the PD-1 expression in cervical cancers documented in recent studies [55,56,57], providing a rationale for immunotherapy that included multimodality treatment. Several studies about recurrent or metastatic cervical cancers are active (NCT03192059, NCT03277482, NCT03452332, and NCT03614949). Our previous study indicated that the D90 < 130Gy was significantly associated with shortened local control and OS, which were less than 40% and 20% at 24 months after RISI [16]. Compared with a previous study, the addition of a PD-1 inhibitor may lead to the superior LC and OS. Meanwhile, a PD-1 inhibitor may also eliminate potential distant metastasis, which was an important pattern of PFS failure.

Moreover, the combination of the PARP inhibitor and radiotherapy may be a promising treatment strategy. As previously mentioned, PARP is an intracellular protein involved in the repair of single and double-stranded DNA breaks [7,8], and radiotherapy exerts its cytotoxic mitotic effects on tumor cells through DNA damage [58,59], so the combination with a PARP inhibitor would sensitize the effect to DNA-damaging induced by radiation. This sensitizing effect has been demonstrated in in vitro studies for both fraction radiotherapy and continuous LDR radiotherapy [60,61,62,63]; meanwhile, some clinical studies have investigated it [64,65].

## 5. Conclusions

PD-1 inhibitor maintenance therapy combined iodine-125 seed implantation may be a promising therapeutic strategy for patients with recurrent cervical cancer.

## Data Availability

Data is contained within the article.
